# Unvaccinated children as community parasites in National Qualitative Study from Turkey

**DOI:** 10.1186/s12889-020-09184-5

**Published:** 2020-07-11

**Authors:** Sıddıka Songül Yalçin, Ayça Gelgeç Bakacak, Osman Topaç

**Affiliations:** 1 Unit of Social Pediatrics, Department of Pediatrics, Faculty of Medicine, Hacettepe University, Ankara, Turkey; 2 Department of Vaccine Studies, Vaccine Institute, Hacettepe University, Ankara, Turkey; 3 Department of Sociology, Hacettepe University, Ankara, Turkey; 4 Ankara Health Directorate, Public Health Presidency, Ankara, Turkey

**Keywords:** Vaccine refusal, Migrants, National vaccine schedule, Dunning-kruger effects, Qualitative

## Abstract

**Background:**

This national qualitative study explores (1) the experiences, observations, and opinions of health care workers (HCWs) about beliefs, socioeconomic, cultural, and environmental characteristics of parents refusing vaccination and (2) regional differences in the identified risk factors; (3) recommended solutions to improve vaccine acceptance in each of 12 regions in Turkey.

**Methods:**

In total, we carried out 14 individual semi-structured in-depth interviews and 10 focus group discussions with 163 HCWs from 36 provinces. A thematic analysis was performed to explore HCWs’ observations about the parents’ decisions to reject vaccination and possible solutions for vaccine advocacy.

**Results:**

Within the analyzed data framework, vaccine refusal statements could be defined as vaccine safety, the necessity of vaccines, assumptions of freedom of choice, health workers’ vaccine hesitancy, lack of information about national vaccination schedule and components, not trusting the health system, anti-vaccine publications in social media and newspapers, and refugees. Suggestions based on the HCWs suggestions can be summarized as interventions including (1) creating visual cards with scientific data on vaccine content and disease prevention and using them in counseling patients, (2) writing the vaccine components in a way understandable to ordinary people, (3) highlighting the national quality control and production in the vaccine box and labels, (4) conducting interviews with community opinion leaders, (5) training anti-vaccine HCWs with insufficient scientific knowledge and (6) reducing the tax of parents whose children are fully and punctually vaccinated.

**Conclusions:**

The solution to vaccine rejection begins with the right approaches to vaccination during pregnancy. Prepared written and visual information notes should present the information as “vaccination acceptance” rather than “vaccination refusal”. Further studies on vaccine refusal rates should be carried out in various regions of the world so that region-specific actions are implemented to decrease the anti-vaxxer movement and to prevent an outbreak of infectious diseases.

## Background

Vaccination is the right of the child as well as a necessity for social life [1]. In recent years, outbreaks of vaccine-preventable diseases have been seen due to increased vaccine rejection and hesitation [2, 3]. The parental vaccination decision is known to be complex and multi-dimensional, being influenced by past experiences, information sources including peers/family, emotions, routine ways of thinking with risk perceptions, trusting the health personnel, and decision-making processes [4–6]. Some factors leading to vaccine refusal may be the same all over the world, such as lack of time, inappropriate behaviors of staff, poor awareness, and fear of adverse events [4, 7–11]. Previous studies have proposed some interventions to overcome vaccine hesitancy, such as parental counseling, improving access to vaccines, implementation of free vaccines, maximizing child health supervision, offering combination vaccines, using electronic medical records, and reminder phone calls to families [10, 11]. However, Turkey’s health care system has some differences to those of other countries. Firstly, the national vaccination calendar is applied free of charge to every child. Therefore, the cost of vaccination is not a reason for missing vaccination. Secondly, family physicians (FP) and their midwives/nurses (family health nurse, FHN) regularly call and remind the family who does not attend the vaccination appointment on time [12]. Thirdly, as most of the family health units are situated so that they are easily reachable by the families, there is no transportation problem. Moreover, the implementation of the national vaccination schedule for infants is an important criterion that is considered when evaluating the FPs’ work performance, and in case of delayed vaccination, a deduction is made from the salary of the FP [13]. In such cases, a missing/incomplete vaccination report explaining the reasons for the vaccine rejection is prepared by the FP, signed by the parents, and notified to the Ministry of Health (MoH). The MoH Vaccination Department examines the report of vaccine rejection and tries to convince the family by phone. The salary deduction of the FP is reimbursed if there is no negligence. Despite these implementations, an increase in cases of vaccine refusal has been observed in recent years in Turkey [14, 15]. The prevalence of unvaccinated children aged 15–26 months increased from 1.6 to 2.9% between the Turkish Demographic Health Surveys (TDHS) of 2008 and 2013 [16, 17]. The ratio in the east of Turkey increased from 2.8 to 3.8%; however, the ratio in the western region went up from 1.6 to 4.7% during the same period.

Previously, it has been stated that pro-vaccine activism should understand the views and behaviors of the parents who do not vaccinate their children [18]. Vaccine acceptance can be improved by identifying the region-specific causes of hesitation/rejection. Turkey is an area connecting Asia and Europe and including Anatolia, an area with a high-density population with people of different ethnic backgrounds, including refugees. The percentages of children aged 24–35 months who did not receive any childhood vaccination were 8% in Syrian refugees and 3.4% in Turkish nationals domestic people, according to the TDHS-2018 [19, 20]. Declining immunization uptake has been reported in many European countries [21, 22]. Identifying the reasons and barriers for vaccine rejection in Anatolia will also help to create universal solution packages in Europe and the world. Qualitative studies are needed in order to show show multifactorial interactions.

Given the increasing refusal rates seen in the national childhood vaccination program in Turkey in recent years, a qualitative study was carried out in health care workers (HCWs) (a) to find out the beliefs, socioeconomic, cultural and environmental risk factors of parents having a role in vaccine refusal in all 12 NUTS (Nomenclature of Territorial Units for Statistics) regions in Turkey; (b) to examine whether identified risk factors vary in different regions; (c) to compile the proposed solutions for the identified risks.

## Method

An interview guide (Table 1) was prepared with a literature review [22, 23]. The data collection began in September 2017 and ended in June 2018. Ten focus group discussions (FGDs) with 163 HCWs and 14 individual semi-structured in-depth interviews were performed. Overall, 36 from 81 provinces were enrolled for the study.

**Table 1:** Outline of the interview and thematic queries

Topic	Thematic Queries
**Necessity of vaccine**	What do you think about the necessity for vaccination of children in terms of individual and public health?
**Causes of vaccine hesitancy**	What are your opinions on the main reasons for the hesitation/refusal of vaccine applications within the national childhood vaccination schedule?
**General characteristics of families who report vaccine hesitancy**	What can you say about the general characteristics of families with vaccine hesitancy/refusal and the barriers to case management?
**Freedom of choice**	Should the family have a choice to vaccinate? How can you evaluate this in the context of child and public health? Why?
**The National Health System**	Are there national health system-related problems in childhood vaccination? If so, what are these? How can these problems be solved?
**Suggestions about how to solve the problem**	How can family physicians and family health workers prevent cases of vaccine rejection? What can be done to resolve vaccine rejection cases in your area?

### Study team

The study team consisted of one social pediatrician (SSY), one sociologist (AGB), and the Head of the Department of Vaccine-Preventable Diseases (OT). OT identified the regions and family health units having vaccine rejection problems and provided the necessary arrangements for the participation of FPs and FHNs working in these units for FGDs. SSY and AGB were not associated with the health care institutions where the study was conducted. SSY and AGB conducted all FGDs, together. Authority officials did not attend the meetings.

### Study participants

The study participants of FGDs were HCWs who were responsible for the vaccine applications, such as Vaccination Follow-up or Evaluation Officer at the Unit for Vaccine Programs of the Department of Public Health Services in the Provincial Health Directorate (Provincial Vaccination Officers), in addition to FPs and FHNs. The board members of expert associations (infectious diseases, family physicians, social pediatricians), academics, and staff responsible for provincial vaccine applications were attended to individual semi-structured in-depth interviews. The purposeful sampling technique with intensity strategy [24], based on the refuser rate of the family health unit in the provinces, was applied. The study included family health units with at least five anti-vaxxer families or the highest rate in selected provinces and districts. FPs and FHNs from these family health units were invited for FGDs.

The sample size was reached on the basis of thematic saturation when all the authors concluded that further interviews were unlikely to provide any new information by evaluating the frequency of new codes with subsequent interviews in each NUTS region.

### Ethics approval

The study instrument and methodology were reviewed and approved by the Ethical Board of Hacettepe University and the Turkish Public Health Institute.

Before participating in the study, participants gave written informed consent after having been informed about the aim of the study, voluntary participation without any imposed obligation, and using a tape to record the interview. People who agreed to tke part in the “In-depth Interviews” and FGDs were asked to fill out a “Data Protection Form” confirming that their data would be kept limited to the primary research only. Their personal information and experience sharing would be anonymized and remain confidential.

The interviews were conducted in an open and non-judgmental manner, akin to empathic neutrality. In face-to-face interviews, participants were asked to give themselves a pseudonym containing the names of the districts they came from (such as the blonde from Meram; the hard-working man from Mamak), and they expressed themselves under these pseudonyms during the interviews.

### Survey periods

#### Phase 1

**Fig. 1:**
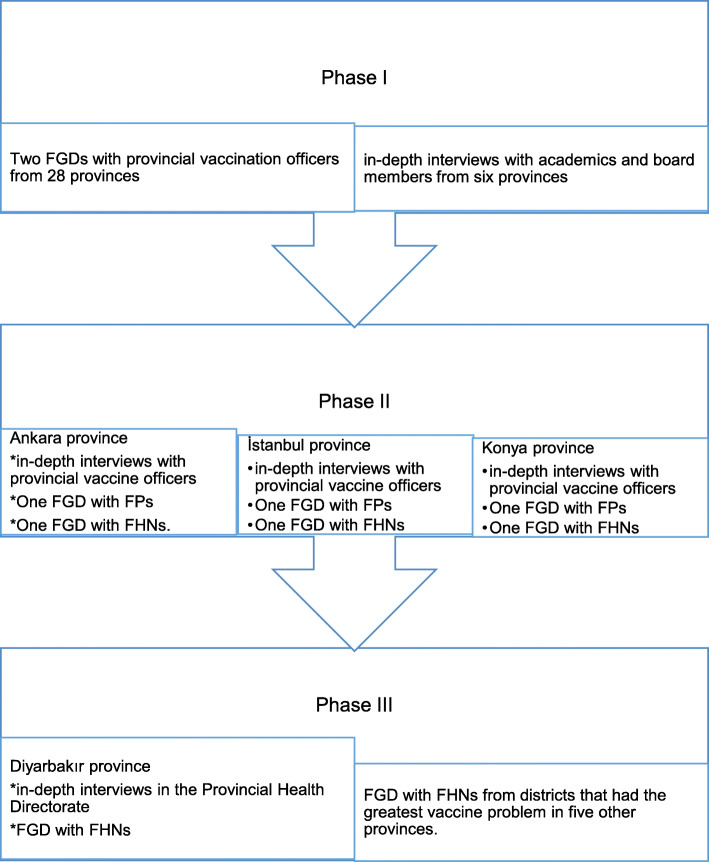
Survey design flowchart

In Ankara on September 21–22, 2017, two FGDs were carried out with provincial vaccination officers who had high provincial vaccine refusal rates or those who had limited vaccine refusal cases but wished to express their opinions. We carried out two FGDs with 15 vaccination officers from 12 provinces and 21 vaccination officers from 16 provinces. Then, in-depth interviews were carried out with three academics and six board members from associations of infectious diseases, and pediatricians or FPs from six provinces on September 23–30 (Fig. 1).

These interviews revealed that most families who rejected the vaccination did not respond to the phone calls from HCWs or other staff from the health care facility of the government. Also, such families did not consent to one-to-one meetings with the professionals designated to these families or even with professionals not associated with their family health unit. Provincial vaccination officers reported that these parents only contacted the FPs and FHNs when their children became ill. It was additionally reported that some families who were hesitant to vaccinate their children were open to discussion, but they resisted vaccination because of the information they had received from the anti-vaccination groups. The FHN was generally found to have the best insight into the structure and dynamics of the families. In the light of obtained information, we planned the next FGDs with both FPs and FHNs in selected districts who had families with vaccine refusal, seeking to identify their problematic families.

#### Phase II

We evaluated the most densely-populated provinces for further interviews. Firstly, we carried out in-depth interviews with provincial vaccine officers in Ankara (October 3, 2017), İstanbul (October 6, 2017), and Konya (March 27, 2018). Later, we held two FGDs in each city. One was performed with FPs and the other with FHNs. Thereby, we conducted two separate FGDs with 12 FPs and 15 FHNs from five districts (Pursaklar, Keçiören, Etimesgut, Yenimahalle, Sincan) in Ankara. We carried out two more separate FGDs with 18 FHNs and 13 FPs from eight districts (Bağcılar, Sultangazi, Arnavutköy, Gazi Osman Paşa, Ümraniye, Küçükçekmece, Başakşehir ve Fatih) in Istanbul. In Konya, 15 FHNs and 14 FPs from three different districts, including Meram, Selçuklu, and Karatay, joined the morning and afternoon sessions, respectively (Fig. 1).

#### Phase III

We carried out two in-depth interviews with the Officer of Vaccination Programs and public health specialists in the Provincial Health Directorate in Diyarbakır (June 4, 2018). Then, two FGDs were conducted with central districts and nearby cities. We conducted the first FGD with 15 FHNs working in six central districts of Diyarbakır, including Sur, Silvan, Kayapınar, Yenişehir, Gaziler, and Ergani. We carried out the second FGD with 20 FHNs from districts that had the most extensive vaccination problem in five provinces including Elazığ, Batman, Muş, Bingöl, Adıyaman (Fig. 1).

### Data analysis

One author (AGB) audio-taped and transcribed the interviews and FGDs verbatim. Then, two authors (AGB and SSY) checked data quality. The data analysis was conducted using thematic analysis techniques on paper [25]: First, data was organized by going back to the interview guide, identifying the questions, and then organizing the data in response to each research question. The second step involved each of the authors independently analyzing the transcripts and the concepts relating to each research question. Third, the concepts were coded into categories. Finally, the categories identified were grouped into overarching themes that answered the research objectives. The authors had subsequent meetings to discuss and agree on the process of data analysis and the reporting of the final themes; that is, they followed a process of external validation of the categories and themes. The analysis was guided by the SAGE Working Group on Vaccine Hesitancy report and a comprehensive literature review [22, 23, 26], based on The Vaccine Confidence Project.

## Results

Among the participants of FGDs, 95% of FHNs, 30% of FPs, and 80% of provincial vaccination officers were female, and between 26 and 52 years old. Eleven of 14 participants of the in-depth interviews were male. In both FGDs and in-depth interviews, the HCWs stated that “parents having vaccine refusal” have become a problem in Turkey over the last 10 years. Almost all staff believed in the necessity of vaccination.

*“Unvaccinated children are ‘community parasites’ and are protected by vaccinated children. But in an outbreak, they will get sick first.” (expert, Ankara).*

*“Unvaccinated child resemble a bomb: whenever it explodes, epidemics occur, and it takes those who have immunodeficiency and chronic illness. As the numbers of vaccine refusals increase, so does their demolition power” (author, SSY).*

Within the analyzed data framework, vaccine refusal statements of HCWs are given in Table 2.

**Table 2:** Observations of the participants about the concerns of families with vaccine refusal

Topic	*Statements*
**Necessity of Vaccines**	
	*“Senior member of the family says that there was no vaccine in our youth. However, we have grown up as right as rain”.(Şanlıurfa)* *“Disease is more “natural” than a vaccine.” (Şanlıurfa and Diyarbakır)* *“Not all vaccines are necessary.” (Trabzon, Malatya provinces and Mediterranean region)*
**Vaccine Safety**	
Overload the immune system	*“Parents say the number of simultaneous vaccinations is too high and unnecessary. Therefore, the vaccine calendar must be rearranged.”(Istanbul)*
Vaccine additives	*“It is said that vaccines include monkey blood, mercury, and pig blood.” (Istanbul, Bolu, Düzce, Gümüşhane, Kütahya).*
May make the child sick (SSPE, autism, cancer) and any adverse reaction after the previous vaccination	*“Increased SSPE incidence in the region causes definite vaccine rejection in the affected families and also in other families in that region. The fact that SSPE cases are not related to vaccination is not fully explained; in 1990–2000, increased cases were linked to the vaccine, the effects of which still continue.”(Diyarbakır)* *“there is a teacher, his child got SSPE …, and he scares other teachers and families with erroneous non-scientific information, as a result of which both childhood and infancy vaccination rates in that district are reduced.”* *“I have doubts about vaccination, too. In one family, there is a vaccinated child who has recently been diagnosed with leukemia; I have hesitations for that reason. My child is vaccinated, but I have doubts about how much of a protective effect it has.”(nurse, Diyarbakır)*
Believing that vaccine changes genetic codes because of additives	*“As vaccines come from abroad, families think their children would be retarded if vaccinated, and they believe the countries that produce vaccines do not vaccinate their own children who will take the country forward.” (Konya)* *“It is said that there is pork blood in the vaccine; it’s therefore haram, and the most important fact is that it makes our children infertile; they do not want to use any birth control either.” (Bingöl)* *“It is said that you will sterilize us with the vaccines.” (Diyarbakır)*
**HCWs’ vaccine hesitancy and misinformation**	
Negative information of HCWs regarding vaccination	*“There’s a professor, a pediatrician, at the university. He says ‘do not vaccinate your child, a child should not be vaccinated for the first two years of his/her life.’ Moms trust him, not me.” (İstanbul)* *“In particular, negative attitudes of obstetricians to vaccines during pregnancy cause mothers to take a negative approach the vaccination of their babies.” (Ankara)* *“Obstetricians have an important role in vaccination refusal. Women safely commend themselves to doctors, and they believe that doctors will not misdirect them. Therefore, in the light of the negative information given by obstetricians, women are not vaccinated with tetanus diphtheria toxoids vaccine/tetanus diphtheria toxoids and acellular pertussis vaccine and even hesitate to vaccinate their children.”(Karabük)* *“Women report that their obstetricians say there is no need for vaccination and they perform all necessary initiatives during their follow-up visits.”(İstanbul)*
Self-refusal, anti-vaccine approaches among HCWs	*“The Ministry of Health sent an official document, saing that health workers born between 1980 and 1990 should be vaccinated. Of them, 90% refused, I could not vaccinate them. In the case of the measles outbreak, health workers had refused vaccination. In our field, we are aiming to achieve something in which he does not believe. The man is a health worker; however, he is not vaccinated, and then he tells families ‘go and vaccinate your children’. Above all, those who do this work have to believe in their job.”.(Bayburt)*
**Distrust of some families regarding the National Health System or its components**	
Mistrust of government health authorities	*“In families who are members of some special religious orders, women avoid obstetric care, are not being given pregnancy vaccinations, and some of them evade healthcare and vaccines by giving birth at home. Some religious groups have their own television and radio channels, and members base their health behaviors on the information given in these channels.” (İstanbul)* *“A remarkable proportion of dedicated followers of some religious orders do not trust state hospitals, they prefer to use small private hospitals. These people are avoiding vaccination.” (Yalova)* *“Local people do not trust state hospitals due to their ethnicity, and for that reason they refuse vaccination.” (Diyarbakır)*
Distrust of organized medicine and public health	*“Some remarkable groups are not vaccinated, and do not even benefit from any health services. They prefer alternative medicine, giving birth at home with the help of district midwives or a labor coach. They hide their pregnancy, and they do not enter pregnancy follow-up programs of the family health unit, because of distrust.” (Konya)* *“Some families go to polyclinics belonging to their cults/groups instead of going to public health institutions; they do not trust state programs and are not vaccinated.” (Ankara)*
Distrust of foreign pharmaceutical companies	*“Families state that vaccines were developed by foreign states to make them infertile, so they refuse vaccines and … they do not trust them.” (Bingöl)* *“Refusal reasons indicated state that vaccines are foreign-originated, and there are unsuitable ingredients.” (Yalova, Konya)* *“They refuse vaccines because vaccines are deployed as a biological weapon, and they come from abroad, so they have content which causes genetic code change.” (Istanbul)*
Insufficient control within the country	*“A family said … … .bring us the vaccine label, show the approval and control of Republic of Turkey … …*. *let the vaccine label write the halal statement and commission approval; show me, then I will have my child vaccinated.” (Konya).*
**Ethical, moral or religious reasons**	
	*“Parents say … .. there are some substances which are not suitable for religion, administering a foreign substance to the body from outside is not appropriate for religion, too.”(İstanbul, Bayburt, Konya)* *“Parents state … … ..vaccination is a sin, forbidden by religion.”(Bolu, Trabzon)* *“Families refusing vaccination mention … … .. the vaccine has been invented to disrupt the blood, and it is illicit.”(Gümüşhane, Ankara, Bingöl)* *“Cult leaders in the region do not permit their denominated families to vaccinate their children, and therefore give them anti-vaxxer information.”(Diyarbakır, Bingöl, Konya, Ankara, İstanbul)*
**Social Media Consulting**	
	*“Anti-vaxxer groups share anti-vaccination conversations, stories, publications, broadcasts. Particularly, tweets sent by an eminent television actress cause quite negative effects.”(Karabük)* *“Especially media channels, bloggers, and brochures of anti-vaxxer groups cause insecurity regarding vaccine ingredients.”(Istanbul)* *“There is a book that includes anti-vaxxer articles and is sold online; it is spreading fast, and information in this book popularize anti-vaxxer views.”(İzmir)* *“They read and learn something on their own and ask questions …*. *I could not refute their hypothesis - I could not even answer them.” (HCWs, Ankara)*
**Freedom of Choice**	
Parents have the right to choose whether to immunize their child	“*It is accepted that vaccination is not mandatory due to the verdict confirming that parents have freedom of choice.” (Ankara)* *“A decision of the Constitutional Court, ‘vaccine is an individual right, it does not affect public health,’ accelerated objections of families.” (Amasya)*
**Refugees**	
	*“Some parents do not even know the birth date of their children, and children may be undernourished or born prematurely, so we cannot even calculate their age.”* *“We talk with parents with the help of an interpreter. We cannot get detailed histories of children, we cannot provide the necessary information about the vaccine.”*

### Necessity of vaccines

Of all HCWs, those from seven provinces reported that vaccine refusals mostly occur in families that openly stated that they regarded vaccines as unnecessary to protect the health of their children (Table 3). It was found that families with a traditional societal structure and extended family model often abstain from vaccination with reference to their own childhood. HCWs reported that the anti-vaxxer families asserted that being in an unvaccinated situation does not cause any problem, and that illnesses help children to respond to viruses, so it makes the immune system robust (Table 2). Moreover, HCWs in İstanbul and Mersin stated that some families do not believe vaccines to have any protective qualities. In addition, HCWs mentioned that even some well-educated families do not accept all types of vaccines, believing some vaccines to be unnecessary because the diseases they protect against have been eliminated (Table 3).

**Table 3:** Supposed Causes and Potential Solutions of Vaccine refusals by regions

Regions	Supposed reasons for vaccine refusal	Potential Solutions
**Istanbul**		
	Vaccine additives (pig blood and gelatin, monkey blood etc.) Overload the immune system Adverse reaction after previous vaccinations Vaccines do not work Religious factors; specific religious lifestyle Distrust of foreign pharmaceutical companies Anti-vaccine groups in social media	Initiatives to solve anti-vaccine politics of the sect or community leaders Imposing sanctions on non-vaccination families (financial penalties, inability to benefit from health discounts, premium punishment, inability to use family medicine system, etc.) Prevention of anti-vaxx broadcasting through social media channels and media organs Preparation of pro-vaccination publications approved by the MoH, explaining the contents Training children for vaccine advocacy Creation of common working areas with the Presidency of Religious Affairs Requirement for being fully vaccinated form at the school enrollment of children
**West Marmara**		
	Vaccine additives (thimerosal and aluminum) Possible adverse reaction after previous vaccinations HCWs’ negative information about vaccination Anti-vaccine publications in social media and newspapers	Prevention of anti-vaccine publications in the media Making public information spots Increasing pro-vaccination campaigns and broadcast publications in social media channels
**East Marmara**		
	Possible serious acute and long-term adverse reactions Specific religious lifestyle (sect); vaccine additives is harmful, forbidden by religion Distrust of foreign pharmaceutical companies Distrust of government health authorities Anti-vaccine groups in social media	Preparing a strategy for the leaders of the congregation/cult structures, especially in the region Establishment of vaccination centers to vaccinate and follow adverse events Preparation of short films and public spots containing subliminal messages Development of an action plan with the Ministry of Education for school vaccination
**West Black Sea**		
	Harmful vaccine additives May make the child sick (autism, SSPE and cancer) Parental right to choose whether or not to immunize their child Specific religious lifestyle and beliefs about vaccine and additives Anti-vaccine groups in social media Problematic health literacy Anti-vaccine publications in social media and newspapers	Informing the public about the vaccination and vaccine content by written and visual publications Preventing non-scientific publications Provision of legal regulations (inability to benefit from payments for Social Security Institution, no coverage of health expenses, increasing premium payments) Raising awareness of mothers about vaccines; Use of message systems such as SMS, MMS and social media tools such as twitter, instagram, facebook Adding a training program on the management of vaccine refusal to the curricula of HCWs To ensure the participation of the district imams in persuasion visits
**East Black Sea**		
	Vaccine additives (pig blood and gelatine) Belief that vaccine changes genetic code Doubting the need for some vaccines Anti-vaccination attitudes of some religions Negative attitudes of obstetricians about vaccination Anti-vaccine publications in social media and newspapers	Bringing legal obligations to families who do not allow their children to be vaccinated
**Northeast Anatolia**		
	May make the child sick (autism, SSPE and cancer) Serious acute adverse reactions Religious beliefs about vaccine additives Lack of information among some health workers Problems in the structure of the national health system Distrust of HCWs Distrust of foreign pharmaceutical companies Anti-vaccine publications in social media and newspapers	Collaboration with the Presidency of Religious Affairs in solving vaccination problems Ensuring the employment of health personnel who know the region and know their problems, reducing short-term appointments
**West Anatolia**		
	Anti-vaccine opinions of sect leader: Vaccine additives are not appropriate to religion. Believing that vaccine changes genetic code and causes infertility Parental right to choose whether or not to immunize their child Potential for long-term adverse events Belief that vaccines are unnecessary Lack of required information on vaccine efficacy and ingredients among HCWs Negative attitudes of obstetricians about vaccination Distrust of HCWs or government health authorities Distrust of foreign pharmaceutical companies Anti-vaccine publications in social media	Providing counseling services for cases with vaccine adverse events and vaccine hesitancy and rejection Establishment of operational committees to assess families who refuse vaccination and to develop strategies to ensure persuasion, Providing postgraduate training to ensure consensus among physicians, Health personnel should be fully informed about the vaccines they apply and should use standard informative brochures about the benefits of the vaccine, Providing positive publications about vaccines in the national vaccination schedule on the media, using public spots, Labelling vaccine boxes to indicate that the vaccine has been controlled by MoH and is halal.
**Central Anatolia**		
	May make the child sick (autism, SSPE and cancer) Serious acute adverse reactions Belief that vaccine changes genetic code or causes infertility because of vaccine additives Religious beliefs about immunization Misinformation about vaccine by health workers Distrust of foreign pharmaceutical companies	Health personnel should be fully informed about the vaccines they apply, Public spots
**Central East Anatolia**		
	Belief that vaccine changes genetic code or causes infertility Vaccine additives Serious acute adverse reactions May make the child sick (autism, SSPE and cancer) Not all vaccines are necessary Parental right to choose whether or not to immunize their child Specific religious life (sect) and religious opinions Some HCWs’ inadequate knowledge about vaccine Distrust of health workers or government health authorities Distrust of foreign pharmaceutical companies Anti-vaccine publications in social media	Ensuring that all HCWs are properly informed about the vaccines and their contents, Revision of the curricula of pre-graduate education of health personnel, Use of correct communication techniques in the community (some rejections can be prevented through giving “fatwa” in the region) Giving vaccine information during pregnancy follow-up Information via mobile phones More attention should be paid to the application of cold chains to the area Transition to oral vaccines instead of parenteral vaccines Accelerating the production of domestic vaccines Explaining that the decrease in infant mortality rates in the region is related to vaccination
**Southeast Anatolia**		
	May make the child sick (autism, SSPE and cancer) Belief that vaccine changes genetic code or causes infertility Serious acute adverse reactions Vaccine additives Not believing that vaccine-preventable diseases can be serious. Disease is more “natural” than vaccine Specific religious life, religious beliefs; sin Some HCWs’ misinformation about vaccine Parental right to choose whether or not to immunize their child Distrust of foreign pharmaceutical companies	Adding vaccine application to the positive performance criteria of family physicians Publishing the scientific information to counteract false information Correct information about the causes of SSPE cases in the region Establishing vaccine information channel between obstetricians and family health workers. Development of legal measures against non-vaccination families; e.g., withdrawal of health insurance cover, penal arrangements to be made in premium payments, direct fines, interruption of financial support of individuals receiving conditional cash transfers,
**Mediterranean**		
	Belief that vaccine changes genetic code or causes infertility Serious acute adverse reactions Vaccines do not work Vaccine-preventable diseases have disappeared Anti-vaccine publications in social media	Preparing cards, brochures, magazines and books about the vaccines and their contents for public use
**Aegean**		
	Belief that vaccine changes genetic code or causes infertility Vaccine additives considered not appropriate to religion Specific religious life (sect) Some HCWs’ misinformation about vaccines Distrust to health workers or government health authorities Anti-vaccine publications in social media	Working with sociologists and social workers to determine a solution within the framework of regional features Preparing cards, brochures, magazines and books about the vaccines and their contents for public use Blocking access to anti-vaccine websites Legal regulations

### Vaccine safety

Vaccine safety was mentioned as a reason for vaccine refusal by 30 of 36 provinces; however, the reasons given for the lack of safety differed according to provinces and regions (Table 3). HCWs mentioned that families object to multiple antigen loading, resulting in vaccine hesitancy. Concerns about the vaccine additives were highlighted as a reason for vaccination rejection both in educated families and in families adopting the Islamic religion and sect lifestyles (Table 2). These data were collected from HCWs in 11 NUTS regions (Table 3).

It was stated that some families in six NUTS regions had no confidence in vaccination because of their belief that vaccines might be the cause behind several diseases, including autism and malignancy (Table 2). The age at admission of a child with subacute sclerosing panencephalitis (SPSS) coincided with measles vaccination at primary school age, prompting the family to believe that the vaccine caused this illness. On the other hand, it was thought that the risk of exposing health problems in children who are vaccinated were greater than in children who are not vaccinated due to the substances in the vaccine. Additionally, it was suggested that the vaccine injected into the body makes children more susceptible for many diseases in later years (Table 3).

Families that particularly embrace the strict normative Islamic lifestyle or who are a dedicated follower of a cult in seven NUTS regions were reported to refuse vaccination, stating that the vaccines come from abroad and cause infertility and a change in the genetic codes of the society (Table 2). Also, some stated that vaccines have been claimed to reduce the intelligence capacity of children.

HCWs reported that some families rejected the re-vaccination of their children on the grounds of purported adverse events in connection with previous vaccinations. Post-vaccination reactions including high fever, nausea, etc., in the immediate vicinity of the family often resulted in rejection of vaccinations, even among the families of HCWs (Table 3).

### HCWs’ vaccine hesitancy and misinformation

Another reason for families to refuse vaccination was a perceived negative approach of the health system and some HCWs. Lack of scientific knowledge or misinformation of HCWs on vaccine-preventable diseases are mentioned in eight NUTS regions (Table 3). In 13 provinces, the anti-vaccination attitude among some HCWs, particularly obstetricians, is also reported to be influential in causing families to refuse vaccination. During the course of interviews, it was noted that some HCWs were not vaccinated; therefore, families who discovered this are not vaccinating their children (Table 2).

### Distrust of some families in the National Health System or its components

Another factor that led to the rejection of vaccines was a lack of confidence in the functioning health system in a wide spectrum. Some families generally distrust the *National Health System*, while others do not believe in the necessity of modern medicine. They feel insecure about the implemented health programs. Therefore, they give birth at home, consult with practitioners of alternative medicine, and even refuse the heel lance in some districts (Table 2). HCWs in four NUTS regions reported that parents who belong to certain ethnic groups or have a cult-lifestyle distrust not only vaccination but also any other programs implemented by the MoH (Table 3). They also observed that such families prefer private hospitals or doctors with a similar cult-lifestyle. Furthermore, HCWs stress that these families also distrust legal public schools and other practices.

HCWs in seven NUTS regions reported vaccine refusals due to distrust of multinational pharmaceutical companies (Table 3). Based on the HCWs’ experience, parents distrust vaccines produced abroad, and the fact that there is no vaccine produced by the government is considered a reason for vaccine rejection.

It was also mentioned that families who think that vaccines from abroad are unsafe also believe that vaccines are not controlled sufficiently by health service providers. Some families are reported to demand the control and approval of vaccine ingredients by national authorities. Some even want to see vaccine labels marked “certified halal”.

### Ethical, moral or religious reasons

HCWs reported that some families in 20 provinces refuse vaccination for religious reasons. There were religious concerns about vaccine ingredients in 11 NUTS regions and refusals on the basis of discouragement from a cult-leader or due to a conservative-religious lifestyle in seven NUTS regions (Table 3). Most of these reasons are categorized as faith-based (Table 2).

### Social media consulting

All the FGDs revealed that many social media channels include anti-vaxxer contents (Table 2). Some families that have access to these channels were reported to state vaccine refusal. All interviews revealed that many negative experiences of allegedly vaccination-related symptoms including convulsions, paralysis and allergies are widely circulated through written and visual media, as well as social media. Based on these interviews, negative opinions expressed on social media and the existence of anti-vaxxer groups and bloggers cause harmful effects on families (Table 3).

### Freedom of choice

According to the statements of HCWs, most families who refuse vaccination refer to their right to freedom of choice (Table 3). They expressed the opinion that HCWs have no right to vaccinate the child, on the basis of personal rights and freedoms. Two main views from two different lifestyles are reported on this issue: (1) Some parents practising certain religious or sectarian lifestyles say that parents know what is good for their children. (2) Some educated families argue that families have the right to choose whether or not to vaccinate their children (Table 2). Previously, a court decided that the national vaccine schedule could only be applied with the parents’ consent [27]. HCWs emphasize that parents refuse vaccines by saying that they are the decision-making authority for their children under the court order.

### Refugees

HCWs have been reported that refugees rely on and benefit from free vaccination and health services of Turkey. However, their children may miss vaccines due to a lack of specific residential address. The high birthrate in refugees and loss of the vaccination card by parents may also reduce the possibility of the family providing accurate health information about their children. Furthermore, the absence of a child identification number and birth certificate creates a problem in planning and monitoring the vaccination of the child. Also, they stress that different languages, communication problems and cultural characteristics are among the obstacles to updating vaccinations in refugees (Table 2).

### Health workers’ suggestions for solving the problem

Precautions suggested to manage the anti-vaccination movement differed according to both refusal reasons and studied regions, as shown in Table 3.

## Discussion

In line with previous studies [4, 6, 7, 28], vaccine refusal reasons given by HCPs are summarized as vaccine safety, lack of necessity for vaccination, assumptions of freedom of choice, health workers’ vaccine hesitancy and lack of information about vaccination in the national vaccine schedule, not trusting the national health system, and anti-vaccine publications in social media and newspapers. All these reasons can be rectified through vaccine literacy. Biasio [29] considered vaccine literacy as a tool mediating the transfer of information.

As documented in other societies [4, 7, 21, 30], the concept of vaccine refusal and hesitancy is evident at different educational levels, within all socioeconomic classes and all ethnic origins, in our survey from Turkey. On the other hand, factors affecting this emerging public health issue vary according to the commonality. Perceived risks for vaccine-preventable disease and fear of vaccination-induced adverse events have also been found in other countries [31, 32]. In addition, vaccine distrust is not only an isolated issue but also associated with the preference for alternative medicine and science rejection in other healthcare fields. Previous literature also mentioned adherence to complementary medicine, differences in the usage of other medicines, and the application of topical fluoride, in association with vaccine hesitancy [33–35].

It is known that pregnant women often hesitate to vaccinate and want to discuss vaccine eligibility [36]. In line with our survey, Danchin et al. [37] showed that vaccine concerns and intentions came into existence during pregnancy.

Our study revealed that most HCWs believe in the safety and necessity of vaccines. Similar to a previous review including midwives [38], only a minority of HCWs in Turkey were unsure and reported some physicians expressing doubt.

### Knowledge and advocacy of health care workers

One critical factor creating parental distrust is the lack of technical knowledge of some HCWs, making it difficult to answer queries and concerns about vaccination. A visual information file about vaccines in the national vaccination schedule, the ingredients of the product, how vaccines prevent disease, possible adverse events, and what the consequences may be if vaccination is abandoned, should be prepared for HCWs, especially FHNs, to guide parents during vaccination. Similarly, vaccination education materials covering the topics of “how vaccines work, herd immunity, vaccine safety” were recommended to improve confidence and trust [39]. Unmet information needs of parents can increase vaccine refusal, whereas by making them truly informed of their choices and the benefits of vaccination, vaccination is advocated [21]. Increasing vaccination compliance and vaccination rates of health personnel [40] will also affect community vaccination.

Monitoring of the national maternal vaccination schedule, primarily adult tetanus-diphtheria vaccination in antenatal care, could be added to the performance criteria of obstetricians, providing tax relief to the physician. This would increase the knowledge of obstetricians about vaccination, break down prejudices, and play a role in increasing the acceptance of vaccination by both the doctor and the pregnant woman. Wilson et al. also reported the importance of healthcare professionals’ views on maternal vaccination and the influence of patient-health care professional relationships on maternal vaccination acceptance [41]. It can be speculated that a mother who is vaccinated during pregnancy will better follow her own child’s vaccination schedule.

Pre-graduate and post-graduate curricula should include routine vaccination schedules with some additional material regarding the importance and necessity of vaccines. Curriculum contents should also highlight the drawbacks of not being vaccinated and its the impact on society. Emphasis on vaccine advocacy should be profound, stressing additionally the effects of vaccine refusal on public health (Table 3). FPs and FHNs should also learn communication skills to manage problematic parents.

“*Negative terminology results in confusion, it is better to be positive. Vaccination acceptance should be used instead of vaccination refusal*.” (SSY)

That fact is crucial in terms of demonstrating that vaccine rejection is clearly not unique to ordinary families but also practiced by some HCWs [42]. Therefore, misinformation in HCWs should be checked and corrected on the basis of current scientific data.

HCWs report that due to frequently changing workplaces, the doctors have reduced chances of recognizing problematic families and taking appropriate measures.

### Initiatives relating to families

The MoH should provide a phone line valid 24/7 and a web page that includes current information on vaccination, vaccine ingredients and adverse events. Before the child’s vaccination appointment, SMS messages about the date of vaccination and the importance/necessity of vaccinations can be sent to the parents’ mobile phones. Providing current information to the parents weeks or months before their visit can help to improve their knowledge, while also preventing them from paying attention to false information on social media.

Centers to follow up post-vaccination adverse events and to investigate the reasons for them and then to inform families about the progress could be established. Cases with suspected vaccine side effects should be monitored closely. In addition, siblings of children suffering serious adverse events should also be vaccinated after being evaluated by skilled health personnel at the Provincial Vaccination Center.

It was shown that poor families who receive conditional cash transfers for a child’s health and education generally have them fully immunized. Financial incentives, such as tax reductions, should be developed for families who have complete all childhood vaccinations and health surveillance programs. Financial incentives could work better than financial penalties. In line with this, Helps et al. reported that financial penalties were not an effective policy measure for non-vaccinating families with an increased desire to maintain control over health choices for their children [43]. In fact, these parents were even found to accept income reductions by removing children from early childhood learning and accessing informal childcare arrangements.

Mandatory vaccination can raise conflicting issues on a parent’s right to choose what they consider is in the best interest of their child. Effects of exercising these rights which should be discussed included the fact that the development of herd immunity in the community is threatened and children cannot be protected against serious and preventable diseases [44–46]. Some HCWs recommended the assessment of vaccination cards and implementation of some limitations in school enrollment for children with incomplete vaccination;

*“Children who are not vaccinated endanger the health of my child at school” (board member).*

HCWs reported that some families rejecting vaccination rely on complementary and alternative medicine. Similar to our study, Attwell et al. observed that parents who refuse vaccination due to concerns regarding toxic and contaminated materials viewed alternative medicine as harm-free, natural, and an effective protective strategy for immune systems [47]. Given the epistemic basis of some parents’ decisions, it is very important to increase confidence in vaccination using current scientific data.

HCWs should recognize and understand parent concerns to resolve vaccine refusal. Some HCWs suggested that “having flexibility in the vaccination schedule and more options or control over the timing of vaccinations” might have encouraged families to participate in decision-making and promote freedom of decision.

### Initiatives relating to groups that have different religious beliefs

As discussed in previous reports [48, 49], Some muslim parents in our survey believed vaccines are haram due to blood or tissue contamination from pigs. Interestingly, the Halal Certificate for the Vaccine Industry is recommended by HCWs and their families.

Training sessions should be arranged especially for personnel working in religious affairs and for Imams, in order to highlight the importance of vaccines and their impact on public health. This would help counteract false information regarding any non-religious substances in vaccines.

It is not possible to solve the vaccination problem in families who do not receive any care from the health institution, in pregnant women who fail to attend follow-up visits, and in those who reject heel blood screening. Since these families act in line with the opinion leaders, it is necessary to try to convince the leaders [50, 51]. The opinion and cult leaders should be given better information in order to change the negative attitudes on the subject, since the families in different sectarian structures determine their own healthcare according to the advice of the leaders.

### Initiatives relating to refugees

A high rate of vaccine-preventable diseases has been reported in Syrian refugees [52]. Despite non-payable primary healthcare services and free childhood vaccinations for refugees in Turkey, language barriers and high mobility among unregistered refugees were reported to limit access to completion of the immunization schedule and lead to missed opportunities for health services [53]. Previously, six FGDs with 33 mothers from Moroccan, Turkish, and other ethnic backgrounds revealed that parents perceived a language barrier in understanding the provided information about the National Immunization Program [54]. Preparing information notes in their native language would help them understand healthcare practices. On the other hand, cash transfers are thought to play a significant role in reducing health inequities and tackling the social determinants of health [55]. Transferring conditional cash to refugees during childhood vaccination would be one way to ensure that the family have their children vaccinated and keep their vaccination card safe. Similarly, China employed three main health system strengthening strategies to significantly improve immunization for the migrant population: first, through waivers of immunization fees or immunization insurance, second, through good management of immunization certificates, and third, by paying extra attention to immunization for particular groups of children, including children of migrants [56]. Up-to-date and age-appropriate immunization rates for migrant children were significantly improved by these strengthening strategies in the health system in China.

### Department of Vaccine-Preventable Diseases, MoH

Registration and data analysis with the national identification number of a child can prevent surveillance errors due to repeated rejection of vaccination for the same child. Follow-up of the number of cases with vaccination rejection in each family health unit can ensure early detection of regional case clusters. Each year, 50–100 infants were enrolled and followed up in a family health unit in Turkey [12]. In this study, five cases of vaccine rejection in a family health unit were defined as a “refusal outbreak”, and HCWs working at the center with the refusal outbreak were selected to be called to interview.

It was possible to establish “what-why” scientific data specific to the regions of families where cases of subacute sclerosing panencephalitis (SSPE) are common.

Vaccine packages and information notes should be written so that they can be understood by the public. The box and inlay could indicate that the Turkish MoH has inspected the vaccine and confirm that neither mercury nor porcine products are used as ingredients.

Families should be informed about the importance of vaccination in all regions; however, risk management protocols should be created region-specifically and implemented only in those regions (Table 3). Initiatives not related to the problems of the region lead to confusion and may further increase vaccine rejection. HCWs from the Aegean region recommend studies with sociologists and social workers to determine a solution within the framework of regional features. West Anatolia HCWs recommend the establishment of counseling services and senior committees for vaccination refusal management and the development of strategies. Similarly, the Nigerian government has appointed consultants to provide supportive supervision and technical assistance to health facility staff for routine immunization, focusing on trust building, advocacy, monitoring and evaluation [57].

### Regulations relating to the prevention of infollution (information pollution) on printed-visual media

Inadequate or problematic health literacy skills are associated with increased vaccine refusal. Similarly, “overconfidence”, as a type of Dunning-Kruger effect, showed that many individuals who lacked expertise failed to appraise their knowledge accurately, got less support for mandatory vaccination policies, and viewed the role that medical professionals have in the policymaking process with skepticism [58].

HCWs stress the issue of anti-vaccination posts in the media and its influence on patients. Tomeny et al. studied trends in anti-vaccination beliefs on Twitter for 6 years in the USA and documented anti-vaccine tweets [59]. Monitoring anti-vaccination beliefs on Twitter can be recommended for pediatricians to refute anti-vaccine arguments. In line with previous studies [60], HCWs in our study advised the preparation of public service advertisements and presentations based upon scientific publications about the credibility and contents of childhood vaccinations. HCWs recommended legal penalties for those who publish “tabloid” news not based upon scientific articles. Also, they suggested that bloggers who post unfounded claims and incorrect information on their social media blogs should be controlled (Table 3).

### Ensuring cooperation between institutions

Legal regulations on childhood vaccination existed in Turkey until 2015. Non-vaccination was previously considered child neglect, and the MoH, the provincial organization of the Ministry of Family, Labour and Social Services, and the judicial system had worked as a team. However, in 2015, a court verdict determined that the decision for or against vaccination must be taken by the family [27]. Currently, HCWs are left alone in dealing with anti-vaccination families, and vaccine hesitancy and refusal have both increased in Turkey. The joint work of the MoH, Ministry of Education, and Ministry of Family, Labour and Social Services should be ensured. Some legal arrangements may be required, depending on the percentage of vaccine rejection and the reasons behind it. As reported previously [11], immunization is a shared responsibility involving the community, service providers, and policymakers.

Importantly, since trust is a prerequisite for public acceptance of vaccines, mistrust of HCWs, governments, institutions, and pharmaceutical companies emerged as a significant theme, as reported previously [61]. Collaborative studies might diminish this mistrust.

### Strengths and limitations

This study is the first national report on vaccine refusal in Turkey. Previous publications include surveys conducted on families or doctors, covering only a few hospitals in one or two provinces [10, 14, 62]. Additionally, the populations of these studies included only families already receiving health care or presenting at hospital because of a health problem. As a limitation of our study, the opinions of families about HCWs and vaccines were indirectly reported. Originally, we planned to do a study including FGDs with anti-vaxxer families. Our initial interviews with experts and HCWs showed that families definitely rejecting vaccination do not respond to phone calls from vaccine representatives and are not open to communication. Therefore, interviews with families on a voluntary basis would detect cases of vaccine hesitation but would fail to show the exact features of vaccine rejection. For this reason, FPs and especially FHNs who are responsible for the childhood national vaccination schedule and know the whole structure of the families, formed our working collective. Sampling included family health units with a record of at least five cases of vaccine rejection or those with the highest number of vaccine rejections in that district. In this way, the present study enabled us to observe the real reasons behind vaccine rejections. As a strength, our study examined families from different religions living in Turkey and the situation with regard to refugees.

In our study, we examined the acceptance of vaccines provided to children free of charge as part of the national vaccination calendar. However, payable vaccines (human papillomavirus, rotavirus, seasonal influenza) were not included in the study. The results of our study cannot, therefore, be generalized to private vaccination protocols. The study focused only on childhood vaccination; however, some comments are made about pregnancy and HCW vaccination, and interrelations between them were also emphasized.

## Conclusion

Vaccination refusal can take the form of different vaccination behaviors such as complete refusal, delaying some or all of the vaccines, reducing the number of vaccinations at one time, selective vaccination, or prolonging vaccination intervals. The findings of our study show that the provision of sufficient information, use of cultural communication skills, trust building, and accountability of the HCWs can be considered the cornerstones to improving vaccine acceptance [6, 11, 63].

Preventing vaccine rejection begins with the right approaches to vaccination during pregnancy. At least five cases of vaccine rejection in a family health unit should be considered a “*vaccine rejection outbreak*”, and thus trigger the evaluation of underlying reasons and initiation of regional measures. In the written and visual information materials to be prepared, the focus should be always be on “vaccination acceptance” rather than “vaccination refusal”.

Key opinion-leading expert health professionals should highlight all the benefits of vaccines and the science behind immunization on social media, blogs, and newspapers.

Further studies on vaccine refusal rates and socio-cultural structures should be carried out in various regions of the world to allow region-specific actions to be implemented, thus counteracting the anti-vaxxer movement and preventing disease outbreaks.

## Data Availability

The datasets used and/or analyzed during the current study are available from the corresponding author on reasonable request.

## Abbreviations

FGDs: Focus group discussions
FHN: Family health nurse
FP: Family physician
HCWs: Health care workers
MoH: Ministry of Health
NUTS: Nomenclature of Territorial Units for Statistics
SSPE: Subacute sclerosing panencephalitis
TDHS: Turkish Demographic Health Survey
